# Natural stibnite ore (Sb_2_S_3_) embedded in sulfur-doped carbon sheets: enhanced electrochemical properties as anode for sodium ions storage

**DOI:** 10.1039/c9ra02301a

**Published:** 2019-05-15

**Authors:** Mingxiang Deng, Sijie Li, Wanwan Hong, Yunling Jiang, Wei Xu, Honglei Shuai, Hui Li, Wenlei Wang, Hongshuai Hou, Xiaobo Ji

**Affiliations:** College of Science, Central South University of Forestry and Technology Changsha 410004 China wenlei_wang@hotmail.com; College of Chemistry and Chemical Engineering, Central South University Changsha 410083 China hs-hou@csu.edu.cn

## Abstract

Antimony sulfide (Sb_2_S_3_) has drawn widespread attention as an ideal candidate anode material for sodium-ion batteries (SIBs) due to its high specific capacity of 946 mA h g^−1^ in conversion and alloy reactions. Nevertheless, volume expansion, a common flaw for conversion-alloy type materials during the sodiation and desodiation processes, is bad for the structure of materials and thus obstructs the application of antimony sulfide in energy storage. A common approach to solve this problem is by introducing carbon or other matrices as buffer material. However, the common preparation of Sb_2_S_3_ could result in environmental pollution and excessive energy consumption in most cases. To incorporate green chemistry, natural stibnite ore (Sb_2_S_3_) after modification *via* carbon sheets was applied as a first-hand material in SIBs through a facile and efficient strategy. The unique composites exhibited an outstanding electrochemical performance with a higher reversible capacity, a better rate capability, as well as an excellent cycling stability compared to that of the natural stibnite ore. In short, the study is expected to offer a new approach to improve Sb_2_S_3_ composites as an anode in SIBs and a reference for the development of natural ore as a first-hand material in energy storage.

## Abstract 2

Antimony sulfide (Sb_2_S_3_) has drawn widespread attention as an ideal candidate anode material for sodium-ion batteries (SIBs) due to its high specific capacity of 946 mA h g^−1^ in conversion and alloy reactions.

## Graphical abstract

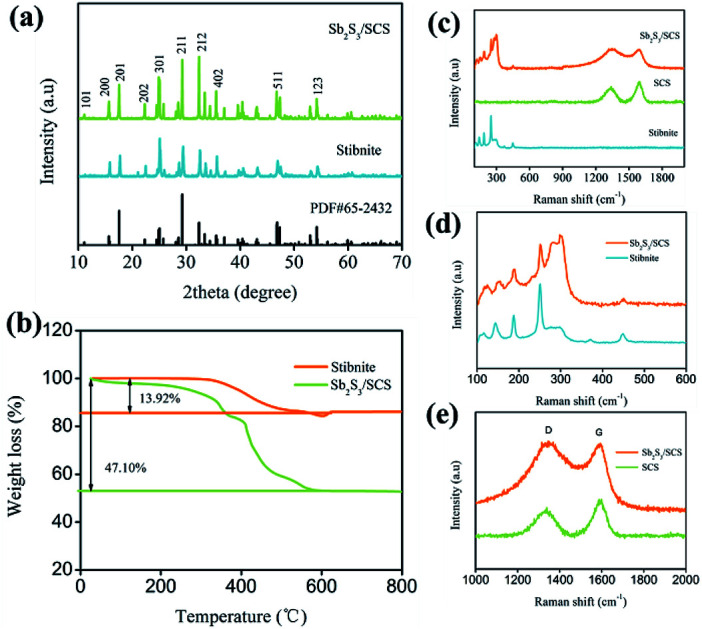

## 1. Introduction

Lithium-ions batteries (LIBs) have received great success in the energy storage domain since their first commercial application.^1^ However, the finite lithium resource can no longer keep pace with the mushroom growth of large scale energy storage systems (ESSs) and electric vehicles (EVs).^2,3^ Recently, sodium-ions batteries (SIBs) were classified as a potential alternative for LIBs owing to a similar operating mechanism to LIBs, abundant sodium resource, and the wide global distribution of sodium.^4^ Unfortunately, the radius of sodium ion is larger than lithium (0.102 nm *vs.* 0.076 nm), which prohibits sodium ion insertion/desertion from active materials.^5,6^ Moreover, typical graphite anode materials that have gained much attention in LIBs exhibit a poor electrochemical activity for sodium ions because the interlayer distance of graphite is bad for sodium ion insertion/desertion.^7^ Hence, it is of much interest to researchers to investigate outstanding anode materials for SIBs with the following features: (1) a high initial coulombic efficiency to enhance the operating factor of sodium in a full battery, (2) a good rate capability to benefit the high-powered density system, and (3) an excellent cycle stability to reduce the utilization cost.^8,9^

Currently, antimony sulfide (Sb_2_S_3_) has been widely considered as a promising anode material for SIBs. Theoretically, Sb_2_S_3_ can generate a high specific capacity of 946 mA h g^−1^ through a conversion and alloying reaction (eqn (1) and (2)), corresponding to 12 moles of sodium and electrons per unit formula in the reaction.^10,11^ The potential advantages of Sb_2_S_3_ include a higher theoretical capacity compared to that of Sb (660 mA h g^−1^) because the lower weight of the S atoms improves the mechanical stability of Sb_2_S_3_ due to smaller volume change from the sulphides during charge/discharge.^12^ Nevertheless, there is an inherent shortcoming that involves the inevitable volume fluctuation (390%) occurring in the sodiation and desodiation process. This fluctuation ruins the structure of the materials, which caused cycle inability.^8^ Therefore, it is crucial to alleviate the materials volume change for the enhancement performance of Sb_2_S_3_ in SIBs. To mitigate the mechanical stress during the sodiation and desodiation process, there is a comprehensively adopted strategy that requires the active substances to combine with a carbonaceous material such as graphene,^13^ RGO,^14^ carbon nanotubes,^15^ carbon spheres,^16^ carbon fibers,^17^ or other substrate materials to fabricate the hybrid composites^18,19^ The functional matrix in the hybrid composites can not only act as a buffer to relieve the volume expansion and restrict the agglomeration of the active substance but also serve as the conducting medium to promote sodium ions transport and electron transfer.^11^

1 Sb_2_S_3_ + 6Na^+^ + 6e^−^ → 2Sb + 3Na_2_S

2 2Sb + 6Na^+^ + 6e^−^ → 2Na_3_Sb

To the best of our knowledge, the majority of Sb_2_S_3_ materials are prepared by chemical methods such as the solvothermal process,^20^ the hydrothermal reaction,^21^ vacuum thermal evaporation,^22^ high-energy ball milling,^23^ or the sonochemical method.^24^ Clearly, these approaches are commonly complicated with low output and by-product contamination to certain extent. Stibnite, primarily a Sb_2_S_3_ ore that belongs to the orthorhombic system (*a*_0_ = 11.20 Å, *b*_0_ = 11.28 Å, and *c*_0_ = 3.83 Å), has been found as huge crystals and a crystal cluster (chemical purity: 99% Sb_2_O_3_) in many areas around the globe.^25,26^ In addition, stibnite is a significant raw mineral material used to obtain antimony and antimony compounds through the roasting reduction method, electrolysis in a strong acid or alkaline solution, and multiple volatilization smelting. Nevertheless, it is certain that the aforesaid methods have high energy consumption, high time consuming, and high pollution levels, which are contrary to green chemistry. If the natural stibnite were treated as the first-hand anode materials for SIBs, researchers would not only take full advantage of the raw materials without many intermediate processes, but would also be able to lessen the negative impacts associated with preparing the electrode such as environmental contamination, high cost, and by-product generation. In general, natural pure minerals have unfavorable electrochemical properties.^27^ Hence, it is necessary to improve the electrochemical performance of natural minerals as green resources to be applicable in SIBs.

For the purpose of green and environment-friendly, we herein report a facile chemical method to prepare a high-performance stibnite/sulfur-doped carbon sheet (Sb_2_S_3_/SCS) electrode for sodium storage by anchoring natural stibnite on sulfur-doped graphitic carbon sheets. Moreover, the introduction of the carbon sheets could evidently relieve the volume variation of stibnite and improved the conductivity of electrode. Finally, the Sb_2_S_3_/SCS composite exhibited exceptional electrochemical behaviors to pure stibnite in SIBs, indicating the potentials of the mineral in energy system applications. Herein, this study can serve as a reference that natural mineral can be treated as first-hand materials in the energy storage domain and can be used to promote the development of antimony sulfide with green and low energy consumption.

## 2. Experimental section

### Preparation of SCS and Sb_2_S_3_/SCS

All regents were purchased and used without further purification. The SCS synthesis was as follows: typically, 0.2 mol of NaOH was blended with 40 mL of acetaldehyde (40%) under magnetic stirring at room temperature. After 1 h, the mixed colorless solution turned yellowish and generated brownish red solids. Then, the reaction vessel was sealed and stored for 120 h. Subsequently, some products were washed under neutral conditions with deionized water and dried at 50 °C for 24 h. Finally, the as-obtained precursor and sodium dodecyl sulfate (SDS) were mixed in proportions (precursor : SDS = 1 : 10 in weight) and annealed at 800 °C for 2 h under an Ar atmosphere with a heating rate of 10 °C min^−1^. The Sb_2_S_3_/SCS composites were prepared as follows: 0.1 g of SCS was dispersed in 20 mL of deionized water under ultrasonication for 1 h, henceforth referred to as A solution. Subsequently, 0.2 g of stibnite (Sb_2_S_3_ ≥ 99%) was dissolved in 20 mL of a Na_2_S solution (0.25 mol L^−1^) under magnetic stirring, henceforth referred to as B solution. Then, the A solution was mixed with the B solution under ultrasonication for 1 h, followed by the addition of 30 mL of a H_2_SO_4_ solution (0.4 mol L^−1^). The obtained brown solid was filtered and washed to its neutral state. Finally, the end products were obtained by annealing at 300 °C for 2 h under an Ar atmosphere with a heating rate of 3 °C min^−1^.

### Materials characterizations

Raman spectroscopy (Renishaw inVia, UK), X-ray photoelectron spectroscopy (XPS a K-alpha 1063), scanning electron microscopy (SEM, FEI Quanta 200) with an energy dispersive spectrometer (EDS), transmission electron microscopy (TEM, JEM-2100F), X-ray diffraction (XRD, Bruker D8 diffractometer Cu Kα radiation with a wavelength of 0.1542 nm), and thermogravimetric analysis (TGA, NETZSCH STA449F3) were used to characterize the morphology and composition of the composites.

### Electrochemical measurements

The electrochemical performances were characterized using CR2016-type half-cells with sodium metal as the counter electrode and Celgard 2400 as the polypropylene separator which were assembled in an MBraun glovebox under an Ar atmosphere. The slurry consisted of 70 wt% Sb_2_S_3_/SCS composites, 15 wt% super P, and 15 wt% carboxymethyl cellulose (CMC) dissolved in deionized water. Subsequently, the obtained uniform slurry was cast on Cu foil, followed by drying at 70 °C for 12 h which was utilized as the working electrode. The mass loading for the active materials was in the range of 0.8–1.2 mg cm^−2^. The electrode comprised of 1 M NaClO_4_ in propylene carbonate (PC) with additional 5% fluoroethylene carbonate (FEC). Cyclic voltammetry (CV) measurements were tested at different scan rates with voltages ranging from 0.01 to 2.5 V, and electrochemical impedance spectroscopy (EIS) was tested at 0.01 Hz and 100 kHz using the CHI 660E electrochemical workstation. Galvanostatic cycling and rate performances were conducted with an Arbin battery cycler (BT2000) at different current densities and voltages between 0.01 and 2.5 V (*vs.* Na/Na^+^).

## 3. Results and discussion

**Fig. 1:**
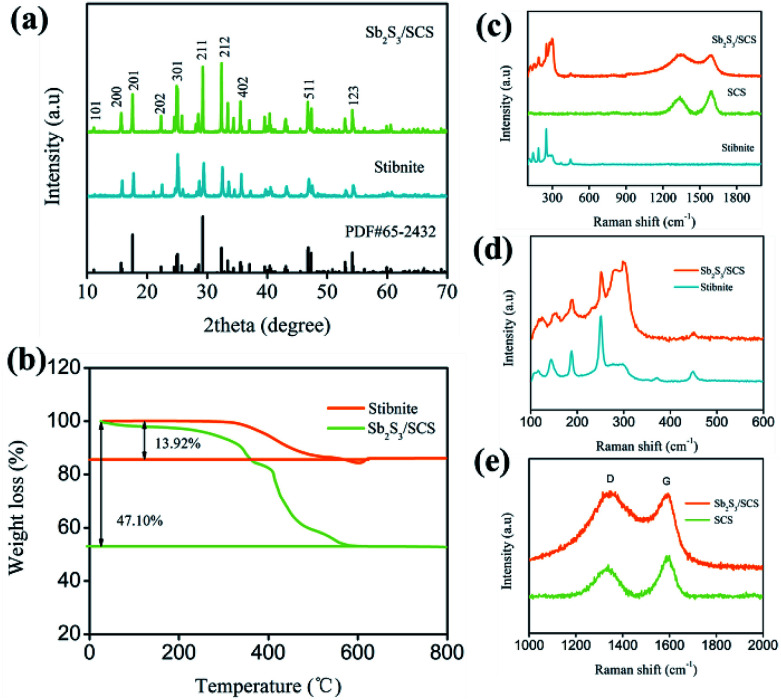
(a) XRD patterns of Sb_2_S_3_/SCS and stibnite. (b) TGA curves of Sb_2_S_3_/SCS and stibnite. (c–e) Raman spectra of Sb_2_S_3_/SCS and stibnite.

To determine the chemical composition and structure, stibnite and Sb_2_S_3_/SCS composites were characterized with X-ray diffraction (XRD). As illustrated in Fig. 1, all the observed peaks for stibnite and Sb_2_S_3_/SCS were indexed to the orthorhombic phase of antimony sulfide (PDF# 65-2432, *Pnma* (62), *a* = 1.130 nm, *b* = 0.383 nm, and *c* = 1.12 nm). The diffraction peaks were located at around 11.15°, 15.69°, 22.31°, 17.67°, 24.91°, 29.16°, 32.29°, 35.70°, 46.62°, and 54.28°, which belonged to the (101), (200), (202), (201), (301), (211), (212), (402), (511), and (123) crystal planes of Sb_2_S_3_, respectively. No other impurity peaks were found, clearly indicating the high purity of stibnite and the Sb_2_S_3_/SCS composites. The peaks for the Sb_2_S_3_/SCS composites were sharp and pointy, indicating a good crystalline nature. In addition, the sulfur-doped carbon sheets could have been amorphous because no extra carbon diffraction peaks were found in the results for Sb_2_S_3_/SCS.^28^ Thermogravimetric analysis (TG) was utilized to quantify the Sb_2_S_3_ content in the Sb_2_S_3_/SCS composite under an air atmosphere. As displayed in Fig. 1b, the mass loss at 700 °C occurred through the conversion reaction from Sb_2_S_3_ to Sb_2_O_4_ and the combustion of the carbon matrix in the air.^16,29^ Consequently, the content of Sb_2_S_3_ in the Sb_2_S_3_/SCS composite was determined to be 66.82 wt%.

The Raman spectra of stibnite, SCS, and Sb_2_S_3_/SCS are depicted in Fig. 1c. The characteristic peaks of antimony sulfide located between 100 to 500 cm^−1^ and two evident peaks in the range between 1200 and 1600 cm^−1^ were related to the D-band and G-band of the carbon materials. For the metal sulfides, the Raman shifts for the metal–sulfur bond were mostly located at the low frequency region (≦500 cm^−1^), which agreed with the special metal sulfides.^30^ In the low frequency range from 100 to 500 cm^−1^ (Fig. 1d), these characteristic peaks for both Sb_2_S_3_/SCS and stibnite were at 121, 151, 188, 249, 301, and 447 cm^−1^, which were mainly ascribed to the S–Sb stretching vibration and S–Sb–S bending vibration.^31^ The characteristic peaks at 121, 151, and 188 cm^−1^ came from the S–Sb–S bending vibration and the bands at 249 cm^−1^ were assigned to the S–Sb stretching, indicating its well-crystallined nature.^32^ The presence of the peak at 301 cm^−1^ could be explained *via* a symmetric vibration from the pyramidal SbS_3_ unit, which had a *C*_3v_ symmetric form.^10^ A weak peak at 447 cm^−1^ could have arisen from the symmetric stretching of Sb–S–S–Sb.^23^ In the high frequency region from 1200 to 1600 cm^−1^ (Fig. 1e), two peaks at 1332 and 1590 cm^−1^ were attributed to the D-band, which were characteristic peaks of the C_sp^2^_ hybrid orbital because of the G-band, a disorder arrangement of the carbon atom, or lattice defects, suggesting an ordered lattice structure from the vibration of C_sp^2^_ in the plane.^33^ According to the *I*_D_/*I*_G_ ratio (0.621), the SCS had many defects and vacancies, which contributed to the diffusion of ions and supported additional reaction sites.^34^

**Fig. 2:**
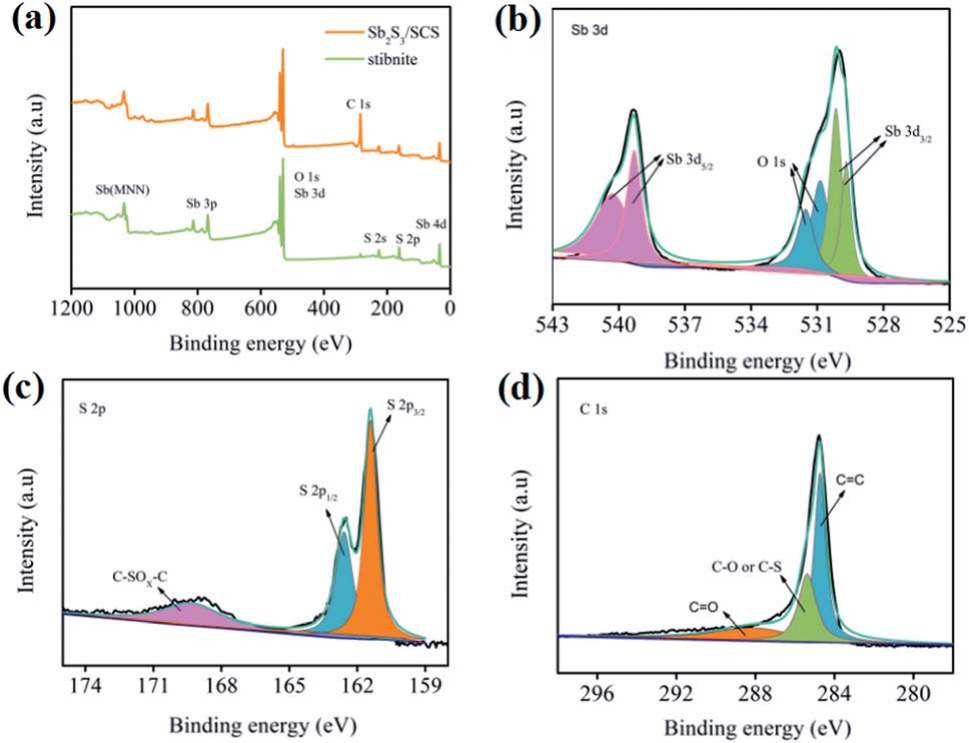
(a) XPS survey of Sb_2_S_3_/SCS and stibnite. The corresponding high-resolution spectra of Sb (b), S (c), and C (d).

X-ray photoelectron spectroscopy (XPS) was utilized to investigate the chemical composition and surface electronic states of Sb_2_S_3_/SCS, as illustrated in Fig. 2a–d. According to the survey spectrum of Sb_2_S_3_/SCS in Fig. 2a, where Sb, S, O, and C were noted, an O element may have come from the oxygen-containing functional groups that were beneficial to the surface redox reaction with Na^+^ on SCS.^35,36^ The high-resolution spectrum of Sb 3d and O 1s is depicted in Fig. 2b. The peaks for Sb 3d_3/2_ (539.2 and 540.1 eV) and Sb 3d_5/2_ (529.6 and 530.1 eV) were associated with the existence of Sb^3+^. The two peaks for O 1s were assigned to C=O (530.8 eV) and C–O (531.5 eV), respectively.^37^ Notably, the S 2p spectrum in Fig. 2c displayed the peaks at 161.3 and 162.5 eV, which were attributed to S 2p_3/2_ and S 2p_1/2_, suggesting a single doublet from the S–Sb bonds. A wide peak at 169.3 eV that belonged to the C–SO_*x*_–C bond revealed that sulfur was successfully doped into the carbon matrix.^4,11,38^ As shown in Fig. 2d, the peaks at 284.7, 285.3, and 288.4 eV corresponded to the C=C, C–O/C–S and C=O bonds, respectively, confirming the existence of oxygen-containing functional groups.^39^

**Fig. 3:**
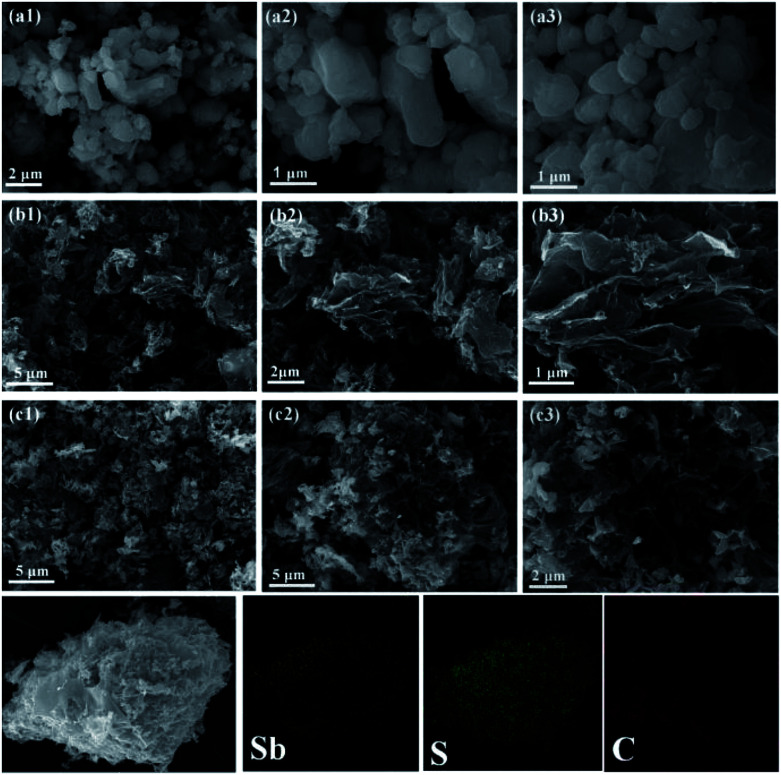
SEM image of stibnite (a), sulfur-doped carbon sheets (b), and the Sb_2_S_3_/SCS composites (c).

**Fig. 4:**
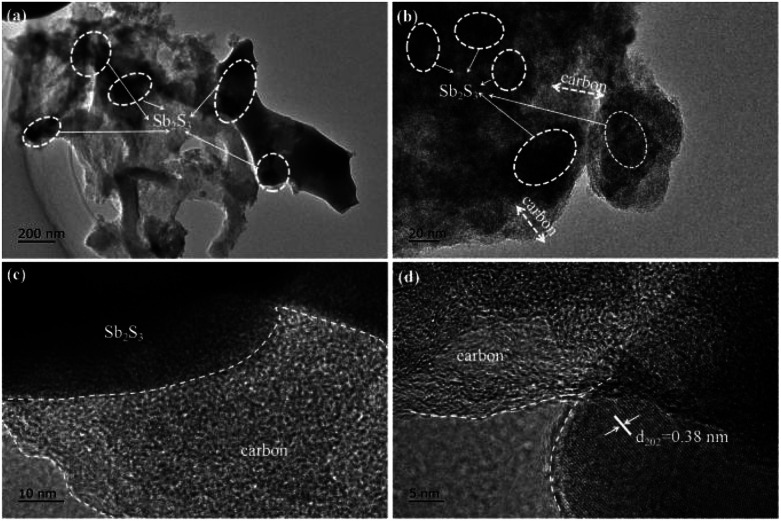
TEM (a and b) and HR-TEM (c and d) of the Sb_2_S_3_/SCS composites.

In order to investigate the morphological characteristics of the stibnite mineral, SCS, and Sb_2_S_3_/SCS, scanning electron microscopy (SEM) was performed. As shown in Fig. 3a, the stibnite mineral did not obviously exhibit any special morphology and had unevenly sized particles. In Fig. 3b, SCS showed a wrinkled and slightly frizzy plate-like structure, which was mutually connected with other sheets. Notably, the special plate-like and connected structure could enhance the electronic conductivity of the sodium ions and shorten its diffusion distance. In Fig. 3c, the stibnite mineral was uniformly decorated on the sheets, and the corresponding elemental mapping images showed that Sb, S, and C were evenly distributed in the Sb_2_S_3_/SCS composites. Transmission electron microscopy (TEM) was performed to further investigate the detailed morphology and crystal structure of the Sb_2_S_3_/SCS composite. As illustrated in Fig. 4a–d, the Sb_2_S_3_ particles were well-dispersed and embedded in the carbon sheets. These results were combined with the results from SEM. Furthermore, a lattice rim with an interplanar spacing of 0.38 nm was found, corresponding to the (202) plane of stibnite, which is consistent with the XRD analysis.^23^

**Fig. 5:**
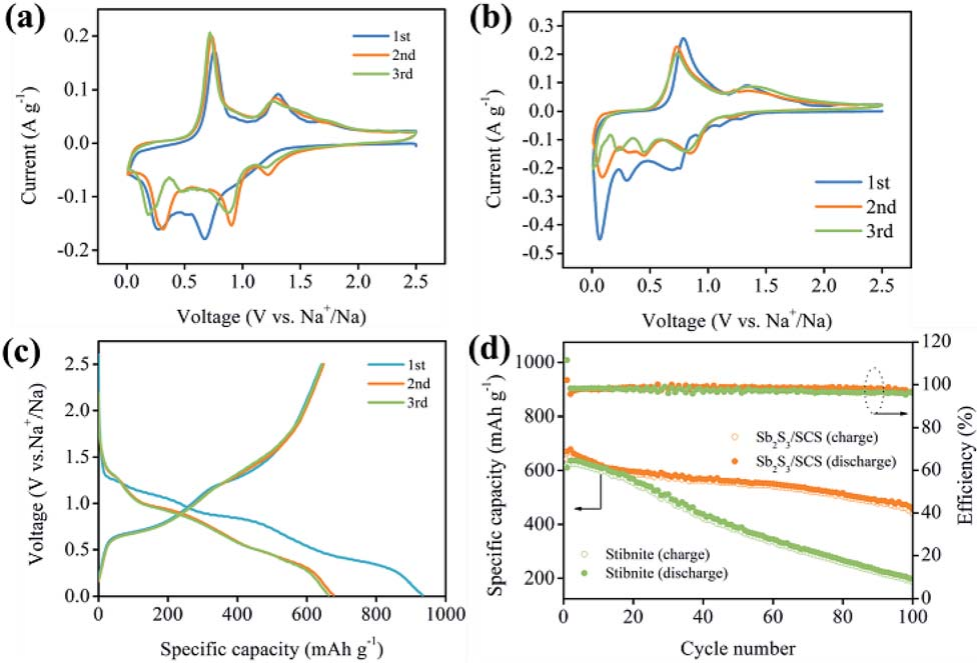
Cyclic voltammograms of the initial three cycles for Sb_2_S_3_/SCS (a) and stibnite (b), galvanostatic discharge/charge curves for Sb_2_S_3_/SCS (c), cycling performance and coulombic efficiencies of Sb_2_S_3_/SCS and stibnite (d).

The electrochemical properties of the Sb_2_S_3_/SCS composites and stibnite ore were studied as SIBs anode by cyclic voltammetry in the voltage range of 0.01–2.5 V. As depicted in Fig. 5a, the first cathodic scan exhibited two current peaks located at 0.67 V and 0.26 V, which were ascribed to the conversion reaction with the sulfur atoms in material (eqn (1)), the alloying reaction of Sb with Na (eqn (2)), and the decomposition to form the solid electrolyte interface (SEI) on the surface of the electrode, respectively.^16^ In subsequent cathodic scans, the location of the peaks was different, which could be explained by the formation of SEI and other irreversible reactions in the first cycle. Moreover, the area of the first scan was larger than that of the subsequent cycles, indicating that the initial discharge capacity was higher than succeeding cycles.^40,41^ During the anodic scan, the peaks at 0.74 V and 1.31 V were assigned to the dealloying reaction (2Na_3_Sb → 2Sb + 6Na^+^ + 6e^−^) and the formation of Sb_2_S_3_ (2Sb + 3Na_2_S → Sb_2_S_3_ + 6Na^+^ + 6e^−^),respectively. As displayed in Fig. 5b, the CV curves of stibnite were different from those of Sb_2_S_3_/SCS, which further indicated the high irreversible capacity loss of stibnite in the cycling process.^11^ Moreover, the reactions between stibnite and sodium during the discharge/charge were as follows:^42^

During discharge:Sb_2_S_3_ → Na_*x*_S + Sb → Na_2_S + Sb → Na_2_S + Na_3_Sb

During charge:Na_2_S + Na_3_Sb → Na_2_S + Sb → Sb_2_S_3_

The galvanostatic cycling curves of the Sb_2_S_3_/SCS composites in the voltage range of 0.01–2.5 V at a current density of 0.1 A g^−1^ are depicted in Fig. 5c. There were two smooth voltage plateaus at 1.08 to 0.86 V and 0.77 to 0.30 V in the first discharge profile, corresponding to the reductive transformation and alloying reactions, respectively. In the charge profiles, the voltage plateaus at around 0.61 to 1.13 V and 1.24 to 1.56 V corresponded to the dealloying reactions and desodiation reactions, respectively. Moreover, the plateau regions of the discharge/charge process agreed well with the related CV curves.

The cycling stability of the electrode materials was quite significant for the application of the batteries. The sodium ion storage performances of Sb_2_S_3_/SCS and stibnite were studied *via* the sodium ion extraction and insertion at a stable current density of 0.1 A g^−1^ in the voltage range of 0.01 to 2.5 V. Benefiting from the introduction of SCS, Sb_2_S_3_/SCS and stibnite showed completely different electrochemical performances. As depicted in Fig. 5d, Sb_2_S_3_/SCS delivered an initial charge and discharge capacities of 642.8 and 934 mA h g^−1^, respectively, with a coulombic efficiency (CE) of 68.82%. However, stibnite delivered an initial charge and discharge capacities of 617.8 and 1008.3 mA h g^−1^, respectively, with a lower CE of 61.27%, indicating that the conversion and alloying reaction efficiency of Sb_2_S_3_/SCS were more effective than that of stibnite.^16^ Moreover, it is a known phenomenon that the initial coulombic efficiency is low in conversion-alloying type materials.^18^ The flaw of the irreversible capacity could be ascribed to the formation of a solid electrolyte interface (SEI) layer owing to the decomposition of the organic electrolyte at low voltage and the side reactions of the oxygen-containing functional groups on the surface of SCS with sodium ions.^11^ Apparently, coulombic efficiency in following cycles was improved evidently through a stable SEI, which might have alleviated the irreversible reactions.^43^ Encouragingly, the reversible capacity of Sb_2_S_3_/SCS still remained 455.8 mA h g^−1^. The capacity retention was 70.8% after 100 cycles, which were much better than stibnite with a reversible capacity of 190.1 mA h g^−1^ and a capacity retention of 30.7%. Evidently, the introduction of the sulfur-doped carbon sheets enhanced the capacity retention and reversibility of the electrode.

**Fig. 6:**
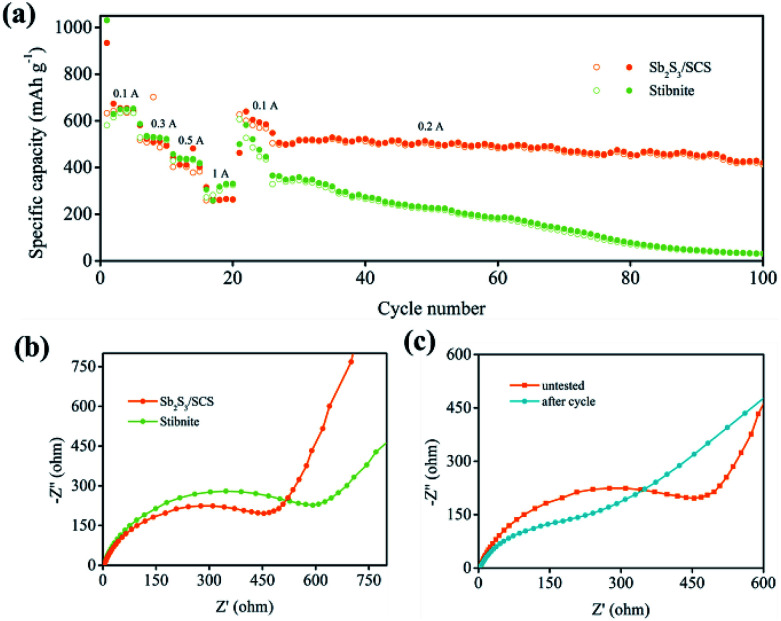
(a) Rate performance of the Sb_2_S_3_ electrode. (b) Nyquist plots of Sb_2_S_3_/SCS and stibnite at the initial state. (c) Nyquist plots of Sb_2_S_3_/SCS at different states.

As depicted in Fig. 6a, the rate performances of Sb_2_S_3_/SCS and stibnite were further investigated at different rates from 0.1 A g^−1^ to 1 A g^−1^. The Sb_2_S_3_/SCS composites exhibited a reversible capacity of 636 mA h g^−1^ every 5 cycles on average. As the current density gradually increased from 0.3 A g^−1^ to 0.5 A g^−1^ to 1 A g^−1^, the related reversible capacity was 497 mA h g^−1^ in the 10^th^ cycle, 392 mA h g^−1^ in the 15^th^ cycle, and 263 mA h g^−1^ in the 20^th^ cycle. A similar capacity fading phenomenon was found for stibnite. In addition, when the current density decreased to 0.1 A g^−1^ after 20 cycles, the reversible capacity of Sb_2_S_3_/SCS recovered to 595 mA h g^−1^ on average. The cycling stability was a key parameter in evaluating the anode materials for applications involving SIBs. Hence, Sb_2_S_3_/SCS was further investigated at a current density of 0.2 A g^−1^ following the rate examination. In the initial cycle (26^th^), the composites delivered a reversible capacity of 509 mA h g^−1^ with a CE of 98%. After 75 cycles, the reversible capacity was 422 mA h g^−1^ with a capacity retention of 82.9%. In contrast, stibnite delivered a much more inferior cycling performance. These results indicated that the content of the carbon sheets in the composites played a prominent role in improving the electrochemical properties and had a positive influence on the rate properties of the materials.

Electrochemical impedance spectroscopy (EIS) was performed to further study the electrochemical performance in the frequency range from 0.01 to 100 kHz at room temperature. The typical Nyquist plot was composed of a depressed semicircle in the medium–high frequency region and a sloping line in the low frequency region. The semicircle at the high frequency region was associated with the formation of a passivation layer and the related impedance between the electrolyte and active materials.^44,45^ Moreover, the charge transfer impedance (*R*_ct_) was illustrated by the semicircle at the medium frequency region, which was treated as a main part of the whole kinetic impedance of the battery and could be computed by utilizing the diameter of the semicircle.^11^ The sloping line at the low frequency region was deeded to the Warburg impedance, corresponding to the diffusion process of the sodium ions in anode materials. As illustrated in Fig. 6b, the Sb_2_S_3_/SCS composites delivered a lower computed *R*_ct_ of 517 Ω compared to that of the stibnite electrode of 668 Ω, suggesting the improved charge transfer of the Sb_2_S_3_/SCS composites at the interface of the electrode and electrolyte. Furthermore, the slope of Sb_2_S_3_/SCS at the low frequency region was more vertical than that of stibnite, suggesting a higher diffusivity of the sodium ions in the electrode.^43^ In Fig. 6c, the initial impedance of Sb_2_S_3_/SCS composites was higher than the cycled electrode of 194 Ω, indicating the activation process and formation of a stable SEI on the surface of the electrode during the insertion/desertion process. The improved diffusivity of the sodium ions and the conductivity of the Sb_2_S_3_/SCS composites benefited from the introduction of carbon sheets, which enhanced the kinetics of the electrochemical process.

## 4. Conclusions

In summary, we successfully prepared Sb_2_S_3_/SCS composites with natural stibnite ore and sulfur doped-carbon sheets *via* an efficient and facile wet chemical approach for sodium ion storage. The composites was analyzed by XRD, Raman, XPS, and other techniques. By applying the Sb_2_S_3_/SCS composites in SIBs, it was observed that the electrode delivered a higher reversible capacity of 455.8 mA h g^−1^ at 0.1 A g^−1^ after 100 cycles, a greater rate performance, and a more exceptional cycling stability than that of the natural stibnite ore. Considering the scalable fabrication method and the idea of green chemistry, we expect that our work can be beneficial to the study of antimony compounds and provide a strategy for natural ore as a first-hand material applied in scale-up energy storage.

## Conflicts of interest

There are no conflicts to declare.

## Supplementary Material
